# Open sesame: Identification of sesame oil and oil soot ink in organic deposits of Tang Dynasty lamps from Astana necropolis in China

**DOI:** 10.1371/journal.pone.0158636

**Published:** 2017-02-24

**Authors:** Anna Shevchenko, Yimin Yang, Andrea Knaust, Jean-Marc Verbavatz, Huijuan Mai, Bo Wang, Changsui Wang, Andrej Shevchenko

**Affiliations:** 1 MPI of Molecular Cell Biology and Genetics, Dresden, Germany; 2 Department of Archaeology and Anthropology, University of Chinese Academy of Sciences, Beijing, PR China; 3 Key Laboratory of Vertebrate Evolution and Human Origins, Institute of Vertebrate Paleontology and Paleoanthropology, Chinese Academy of Sciences, Beijing, PR China; 4 Xinjiang Uygur Autonomous Region Museum, Ürümchi, PR China; New York State Museum, UNITED STATES

## Abstract

Lamp illuminants evidence the exploitation of natural resources, animal and plant domestication, commerce, religious practices and nutrition of ancient populations. However, the physicochemical analysis of their major constituent—burned, degraded and aged mixture of triacylglycerols is imprecise and may lead to ambiguous interpretations. We applied proteomics to analyze fuel deposits from eight lamps dated by 6^th^ to 8^th^ centuries AD that were excavated at the Astana necropolis (Xinjiang, China) and determined their origin by identifying organism-specific proteins. Proteomics evidence corroborated and detailed the assignments of source organism relying upon comparative profiling of intact triacylglycerols by shotgun lipidomics. We found that ruminant (mostly, sheep) fat, cattle ghee and sesame oil were common combustibles in Astana and concluded that sesame as an oilseed appeared in China under Tang Dynasty concomitantly with the expansion of Buddhism.

## Introduction

Since prehistory, artificial illumination has been an essential factor of economic, social and cultural development [1,2]. Lamp pottery, wick materials and fuels reflected local household habits as well as cultural and trade communications. Intact oil lamps containing solid deposits of fuels are among most common artifacts found at ancient settlements and burial sites. Often they were not regarded as objects of fine art and ignored by looters. While the shape, decoration and material of a lamp dish is a common subject of ethnographic and historical interpretations the importance of fuel deposits as a material evidence of cultural, religious and economic practices was recognized only recently.

Identification of ancient fuels is usually based on compositional or isotopic profiling of free fatty acids and triacylglycerols (TAG) [3–7]—major components of animal fats and plant oils. Also the identification of specific molecular markers such as erucic and gondoic acids in *Brassicaceae* plants or isoprenoid fatty acids in some aquatic animal fats [7–10] assists the assignment of organisms of origin. Typically, molecular profiles of fatty acids or TAG acquired by gas or liquid chromatography—mass spectrometry (GC-MS or LC-MS) are screened against a collection of representative profiles of contemporary oils and fats chosen according to ethno-geographical considerations. However, in lamp fuels TAG are destroyed by burning, growing microorganisms and aging. Direct identification of ancient plant oils is hampered by rapid degradation of unsaturated fatty acids [5,8,11–13]. Also, common use of mixed fuels and their natural diversity alters the lipid composition in an unpredictable way and increases interpretations ambiguity. The paucity of reliable analytical methods is a major reason why, despite vast diversity of illuminant oils and fats, only a few were identified in ancient lamps [2–4,6–10,14].

Although plant oils or animal fats are not protein-rich, we hypothesized that ancient recipes used for recovering fats from raw plant or animal materials might co-isolate organism-specific proteins in a minor, yet detectable quantities. We applied proteomics together with shotgun profiling of TAG, Fourier-Transform infrared spectrometry (FT-IR), light microscopy and electron microscopy to determine the organismal origin of organic deposits adhered at the inner surface of ancient lamps.

Oil lamps are commonly encountered at archaeological sites in China; however the recovered organic materials have never been systematically characterized. This study is focused on fuels and wicks from eight lamps dated to 6^th^ to 8^th^ century AD and excavated at the Astana Necropolis (Xinjiang, China)–a cemetery associated with an ancient oasis settlement at the Silk Road. Along with the common combustibles, we identified the earliest known specimen of sesame oil and oil soot ink. We concluded that sesame–a major economic crop in contemporary Eastern Eurasia—was known in China as an oilseed already at the beginning of Tang Dynasty.

## Materials and methods

### Location and ethnocultural background of the Astana necropolis (Xinjiang, China)

**Fig 1:**
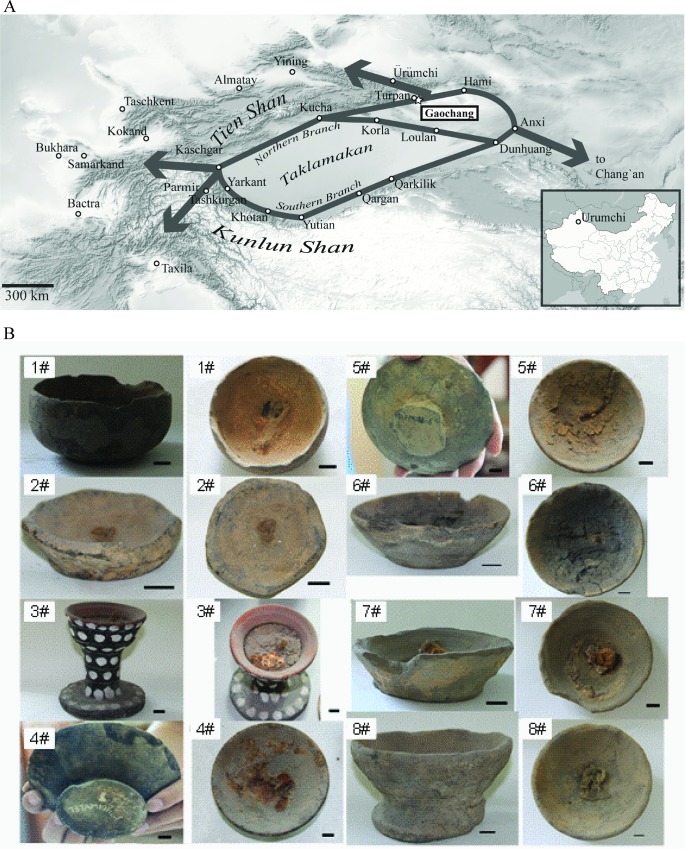
Location of Gaochang and lamps excavated at its Astana necropolis. Panel A: northern and southern branches of the Silk Road bypassing Taklamakan desert [58] and geographical location of Gaochang are shown on an open map from www.opentopomap.org (available under CC BY SA license). Panel B: Side and top views of the eight lamps whose fuel deposits were characterized here. Scale bar: 2 cm.

The necropolis (42.882°N 89.529°E) covers more than 10 km^2^ and is located in Turpan region at the northern rim of Taklamakan desert (Fig 1A). Over more than five centuries (3^rd^ - 8^th^ AD) it served as a public graveyard for the population of ancient oasis settlement Gaochang (1st—14 century AD). Because of its location at the Silk Road junction, Gaochang was a prominent trade hub dominated by Han Chinese migrants [15] under Tang Dynasty (640–803 AD). Dry and hot desert climate of Turpan region supported excellent preservation of funeral artifacts and diverse organic materials including fragments of paper manuscripts, fine silk paintings and food remains.

### Oil lamps excavated at the Astana necropolis

Eight oil lamps examined in this work were excavated at the Astana cemetery in 1964–1973 by Xinjiang Uygur Autonomous Region Museum (Ürümchi, PR China). Seven of them were “bowl”-shaped and dated to Tang Dynasty. Another lamp (pottery 3#) was “dou”–shaped (a circular bowl supported by a stem rising from a flaring base) and dated to the middle of 6^th^ century AD. All lamps contained charred yellowish-brown or black deposits adhered at their inner surface and well preserved (except for 6#) wicks (Fig 1B and Table 1). A few milligrams of ancient organic masses scraped from each lamp were examined by light and electron microscopy, FT-IR spectrometry and then their lipid and protein composition was determined by mass spectrometry.

**Table 1:** Composition of organic deposits in Astana oil lamps.

Lamp	Code^a^	Date*	Proteomic analysis		Lipidomic analysis^c^	Wick	Content attribution
			Groups of identified proteins	Identified proteins (peptides^b^)			
**1#**	64TAM10:7	AD 617–661	1#a: Wick region: seed proteins from *Sesamum indicum* and traces of ruminant collagens	3(8) and 2(9)	*Not sampled*	Cotton and hemp	Oil soot ink made in burnt sesame oil lamp with collagen glue from horse, cow and camel bones
			1#b: Periphery of the dish: collagens, tendon, cartilage and blood proteins from camel, horse, cattle	70(>700)	Adipose fat, multiple matches		
**2#**	64TAM10:9	AD 617–661	Seed proteins from *Sesamum indicum* and *Caprinae* blood proteins	4(37)^d^ and 9(48)	no exact match	Hemp and ramie	Multiple fuels: sesame oil and *Caprinae* adipose fat
**3#**	72TAM169:54	AD 558	Ruminant hemoglobin	1(2)	no exact match	*Not sampled*	Ruminant adipose fat, probably from mixed animal sources
**4#**	73TAM191:21	AD 681	Seed protein from *Sesamum indicum*	1(4)	n.d.	damaged	Sesame oil; burnt
**5#**	73TAM192:: 6	AD 724	Seed proteins from *Sesamum indicum*	3(12)	n.d.	Plant fiber	Sesame oil, burnt
**6#**	72TAM223:16	AD 690–741	Seed proteins from *Sesamum indicum* and traces of ruminant collagens ^e^	4(27) and 6(5–21)^e^	n.d.	burnt	Sesame oil; burnt
**7#**	73TAM504:10	AD 608–698	*Caprinae* blood proteins	3(8)	Adipose fat, multiple matches	Hemp and ramie	Sheep adipose fat
**8#**	73TAM517:3	AD 698	Seed proteins from *Sesamum indicum; Caprinae* blood proteins and cattle milk caseins	7/(126)^d^; 7(33) and 2(6)	Degraded cattle dairy fat	Cotton and ramie	Multiple fuels: sesame oil, *Caprinae* adipose fat and ghee (cattle)

n.d–not detected.

^a^– 64, 72 or 72 indicates the year of excavation (1964, 1972 and 1973); “TAMXXX”:–tomb number according to the excavation records.

^b^—including peptides detected in all repeated analysis of the sample.

^c^—based on profiles of fragments of neutral losses of fatty acid moieties in TAG 44:0 and TAG 42:0.

^d^—apart of *Sesamum indicum* the sample contained a few peptides matched to seed storage proteins from non-oilseed plants (S1 Dataset).

^e^—traces of collagen were not detected at all locations at the lamp surface.

*—according to ancient writings (in Chinese) found in the tomb.

Samples coding (Table 1) is provided according to the excavation records. No permits were required for this study. Analyzed organic deposits are stored in the Department of Archaeology and Anthropology, University of Chinese Academy of Sciences (Yuquanlu 19A, Beijing, P.R. China) and their chemical analysis is a part of Department’s research program.

### Fourier-Transform infrared spectrometry

FR-IR spectrometry was performed on a Nicolet 6700 spectrometer (Thermo Nicolet Corp., Madison, USA). Ancient residues were ground into powder that was mixed and pressed with KBr. Wick fibers were cleaned with absolute ethanol, pressed against the attenuated total reflectance (ATR) crystal ‘window’ and analyzed using 4 cm^-1^ spectral resolution with averaging of 32 scans.

### Proteomic analysis

20-25mg of an organic deposit were scraped from several patches at the inner lamp surface. Dried material was weighted and crashed with a disposable pestle in 1.5ml Eppendorf tube. Proteins were extracted with 2% sodium dodecyl sulfate (SDS) and analyzed by GeLC-MS/MS [16]. Proteins from reference samples (S1 Dataset) were extracted and analyzed in the same way. Proteins were identified by MASCOT v.2.2.04 software (Matrix Sciences Ltd, London, UK) by searching against a comprehensive (all species) NCBI protein sequences database (compiled in May 2014 from 39562230 entries) [16]. False discovery rate (FDR) for peptides whose scores exceeded the identity threshold estimated *via* decoy database search option of MASCOT software, was below 2.3% for all samples. Blank LC-MS/MS runs (*in-gel* digests of void gel slabs) were performed prior to analyses of archaeological and contemporary reference samples. Where specified, sequence similarity searches were performed by MS BLAST program at the MS BLAST web server (http://genetics.bwh.harvard.edu/msblast/). Label-free quantification of the relative abundance of protein groups was performed using Progenesis software (NonLinear Dynamics, Newcastle) as described [17].

### Shotgun lipidomic profiling of TAG

Lipids were extracted from 5 mg of dried archaeological materials or equal amount of contemporary reference samples of oils and fats (S1 Fig) by methyl *tert-*butyl ether (MTBE) [18]. Briefly, to reduce lipid background, prior sample extraction glassware was washed three times with the mixture of methanol / MTBE / water (1:1:1, v/v/v). Solid samples were disintegrated with a pestle into fine powder in a glass vial. Alternatively, 15 μl of oils were taken directly with no pre-treatment. 700 μl of MTBE / methanol mixture (10:3, v/v) were added and incubated in a shaker for 1 hour at 4°C. Then 130 μl of water were added, samples were further incubated in a shaker for another 15 min and centrifuged for 5 min at 13400 rpm and 4°C. 500 μl of the upper MTBE phase were collected into a new glass vial, closed with Teflon liner screw cup and stored under nitrogen at -20°C until analyzed. Control samples were prepared in the same way by adding solvent to empty vials. Prior mass spectrometric analyses, aliquots of lipid extracts were diluted with chloroform / methanol / 2-propanol mixture (1:2:4, v/v/v) containing 7.5 mM ammonium formate: extracts from the archaeological samples were diluted 10-times and extracts of contemporary reference oils or fats 1000-times. Shotgun lipidomics [19] was performed on a Q Exactive instrument (Thermo Fisher Scientific, Bremen, Germany) equipped with a robotic nanoflow ion source TriVersa NanoMate (Advion BioSciences, Ithaca NY) using chips with the diameter of spraying nozzles of 4.1 μm. The ion source was controlled by the Chipsoft 8.3.1 software (Advion BioSciences). Ionization voltage and backpressure were set at +0.96 kV and 1.25 psi, respectively. Temperature of the ion transfer capillary was 200°C; S-lens RF level 50%. FTMS spectra were acquired within the range of *m/z* 200–1200 with the target mass resolution of R_*m/z* 200_ = 140000 and automated gain control (AGC) of 3x10^6^. MS/MS spectra were acquired in *t-*MS2 mode for *m/z* range of 200.5 to 1100.5 with 1 Da step under the following settings: mass resolution of R_*m/z* 200_ = 140 000; AGC of 2x10^4^; one microscan accumulated; Q1 isolation window was set to 1.0 Th; normalized collision energy to 13% with default charge state of 1. TAG ammonium adducts [M+NH_4_]^+^ were identified by LipidXplorer software [20,21] in FTMS spectra with the mass tolerance of 5ppm and signal-to-noise exceeding 3.0. Peaks also detected in control samples were disregarded and not used in further quantification. To determine the relative abundance of fatty acid moieties in isobaric TAG precursors in FT MS/MS spectra LipidXplorer was set to recognize the corresponding neutral loss fragments. Molecular Fragmentation Query Language (MFQL) queries employed by LipidXplorer for interpreting shotgun FTMS and FT MS/MS spectra are available at the LipidXplorer wiki site: https://wiki.mpi-cbg.de/wiki/lipidx/index.php/Main_Page.

Abundances of TAG isobaric species sharing the same number of carbons and double bonds in their fatty acid moieties were normalized to the total abundance of all TAG molecules detected in each sample.

### Electron and light microscopy

Uncoated samples were imaged by SEM using a Magellan 400 FEG-SEM (FEI, Eindhoven, The Netherlands) in immersion mode with accelerating voltages of 3 and 5 kV, beam current 100 pA under variable magnification. For starch grains analysis 20 mg of ground samples were mixed with water and the liquid slurry was examined under Zeiss Axioskop 2 polarizing light microscope. Contemporary oilseeds were delipidated prior analyses [18].

### Fiber analysis

A section of ancient wick was treated with acetic acid and hydrogen peroxide (1:1, v/v) at 60°C for 6 hours. After rinsing with water fibers were examined under Nikon eclipse LV100POL light microscope with 500-fold magnification and compared with contemporary specimen. Drying-twist test was performed as described in [22–24].

## Results

### Light microscopy, electron microscopy and FT-IR spectrometry of organic deposits

Characterization of lamp deposits by electron and light microscopy revealed variable texture of deposited material covered with a dense layer of grown fungi. Importantly microscopy did not recognize plant-specific micro-particles, such as starch grains (S2, S3 and S4 Figs).

Fourier-Transform Infrared spectrometry (FT-IR) of samples 2#, 4# to 8# and 1#a detected signals typical for charred organic materials [25] (S6 Fig). Abundant triacylglycerol-specific signals at 1738 cm^-1^, 1467 cm^-1^, 1161 cm^-1^ and 1097 cm^-1^ in the sample 3# suggested that the lamp was filled with enkindled fat or oil. Materials collected at the edge of the lamp 1# (1#b) showed prominent amide signals at 1650 cm^-1^, 1550 cm^-1^ and 1450 cm^-1^ suggested that the charred material was enriched in proteins.

### Organismal attribution of fat fuels by tandem mass spectrometry of low molecular weight TAG

Next, lipids were extracted from *ca* 5 mg of fuel deposits by methyl-*tert-*butyl ether and extracts subjected to shotgun profiling by high resolution mass spectrometry [19]. In five deposits (1#b, 2#, 3#, 7# and 8#) we detected very similar mass profiles consisting of abundant molecular ions [M+NH_4_]^+^ of triacylglycerols (S2 Dataset). It is known that TAG molecules comprising short (C4 to C10) or unsaturated fatty acid moieties are degraded in aged fats and their molecular profiles are not unequivocally matching profiles of contemporary reference products. Particularly, TAG profiles of aged dairy fats and adipose fats might look very similar and do not allow unequivocal identification of the source organisms without supporting ethnocultural or archaeological evidence, tandem mass spectrometry (MS/MS) [11,26] or compound specific isotope analysis (CSIA) [27]. However, the attribution of Astana lamp fuels by CSIA might be highly ambiguous because of abundant lipids of mold (mostly, fungi of *Aspergillus* genus) that are rich in C16 and C18 fatty acids.

**Fig 2:**
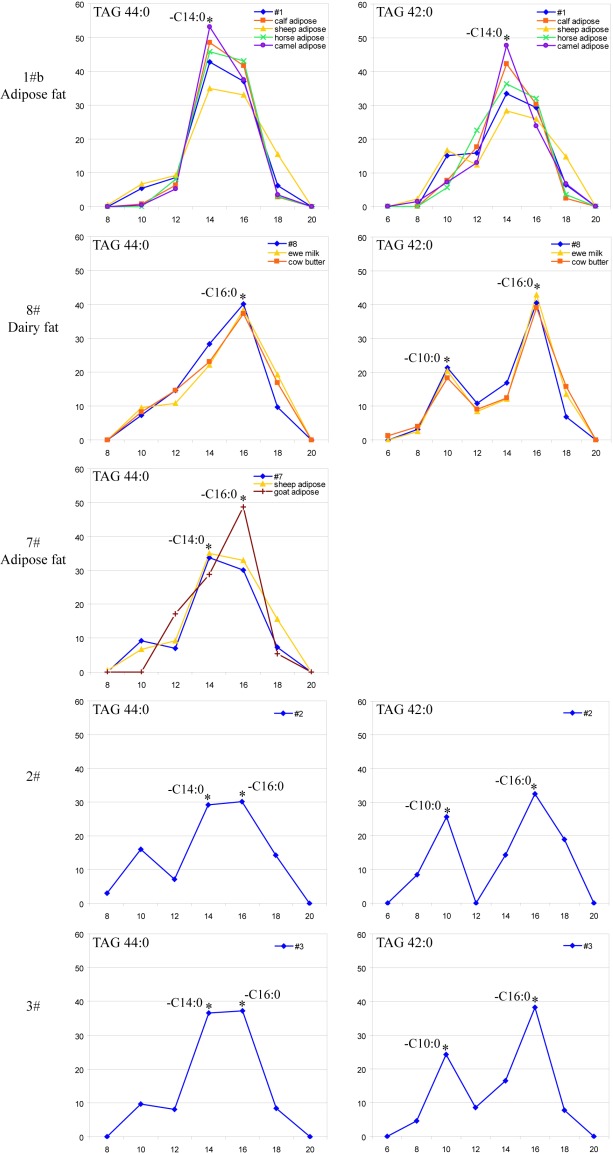
Relative abundance of fatty acid moieties in TAG 44:0 and TAG 42:0 in fuels from five Astana lamps and in contemporary fats. Relative abundances (*y-*axes) were determined by MS/MS fragmentation of ammonium adducts of TAG 42:0 (*m/z* 740.677) and TAG 44:0 (*m/z* 768.708). The number of carbon atoms in each fatty acid moiety is at the *x*-axes. Astana samples are in blue, reference samples—in other colors. Data points are connected for better readability; relative abundances of diagnostic fatty acids C10:0, C14:0 and C16:0 are designated with asterisk.

A TAG molecule consists of three fatty acid moieties attached to the glycerol backbone *via* ester bonds. Isobaric (*i*.*e*. having the identical molecular weight) TAG are composed of different fatty acid moieties. However, they share the same total number of carbon atoms and double bonds. During MS/MS experiment, molecular ions of TAG with the particular *m/z* are isolated by a tandem mass spectrometer and fragmented by colliding with a neutral gas. Upon their collisional activation, fatty acid moieties are dissociated from molecular ions as neutrals. They could be identified by considering mass differences between the *m/z* of molecular ion and corresponding fragments, whose abundance is reflective of the relative content of corresponding fatty acid moieties in co-fragmented precursor ions of isobaric TAG. To determine the organismal origin of fuels, we subjected lipid extracts to shotgun MS/MS and fragmented molecular ions of TAG comprising less than 46 atoms of carbon in their fatty acid moieties [26,28]. Importantly, the low molecular weight TAG are not masked by mold lipids: they are very low abundant in most fungi that typically lack fatty acids with less than 12 carbon atoms [29]. We determined fatty acid compositions of TAG precursors in extracts of Astana fuels and compared them with the composition of the same precursors in 15 lipid extracts from contemporary adipose tissues and dairy products of animals that are believed to be common to households in Turpan area in antiquity (S1 Fig and Fig 2). The similarity of fatty acid profiles suggested that fuel deposits from the lamps 1#b and 7# might contain animal adipose, whereas sample 8# contained a cattle or ewe dairy fat (Fig 2). Fatty acid profiles in 2# and 3# matched no reference extracts. No TAG with unsaturated fatty acid moieties common to plant oils were detected.

### Protein composition of Astana fuels

MS/MS investigation of short-chain TAG suggested a plausible fuel source for three out of the total of eight lamps, however their organismal origin remained ambiguous. Therefore, we set out to identify proteins co-isolated with fats and could serve as organism-specific markers [16,30]. From a separate *ca* 25 mg of fuel deposits we extracted proteins by 2% SDS and analyzed extracts by GeLC-MS/MS [16] (S1 Dataset). Mold proteins, human background proteins [17] and proteins detected in blank SDS PAGE slabs, were disregarded. The remaining proteins from all lamp fuels, with the exception of sample 1#b, fell into two large groups (Table 1). The first group consisted of animal blood proteins, *e*.*g*. hemoglobin and serum albumin. They were identified in fuels from five lamps 1#b, 2#, 3#, 7# and 8#, in which we also detected TAG (Table 1; S1 and S2 Dataset). Blood proteins are common to vessels-traversed adipose tissues [17,31] and, consistently with lipidomics findings (Fig 2), we attributed these fuels to animal fats. Hemoglobins identified in fuels from lamps 1#b and 3# could originate from several *Ruminantia* animals. Sequences of blood proteins from samples 2#, 7# and 8# were unique for *Caprinae* genus and identified peptides equally matched sheep and goat proteins. Since TAG profile in sample 7# did not match the profile from contemporary goat fat (Fig 2, S1 Fig) we attributed it to degraded sheep adipose.

**Fig 3:**
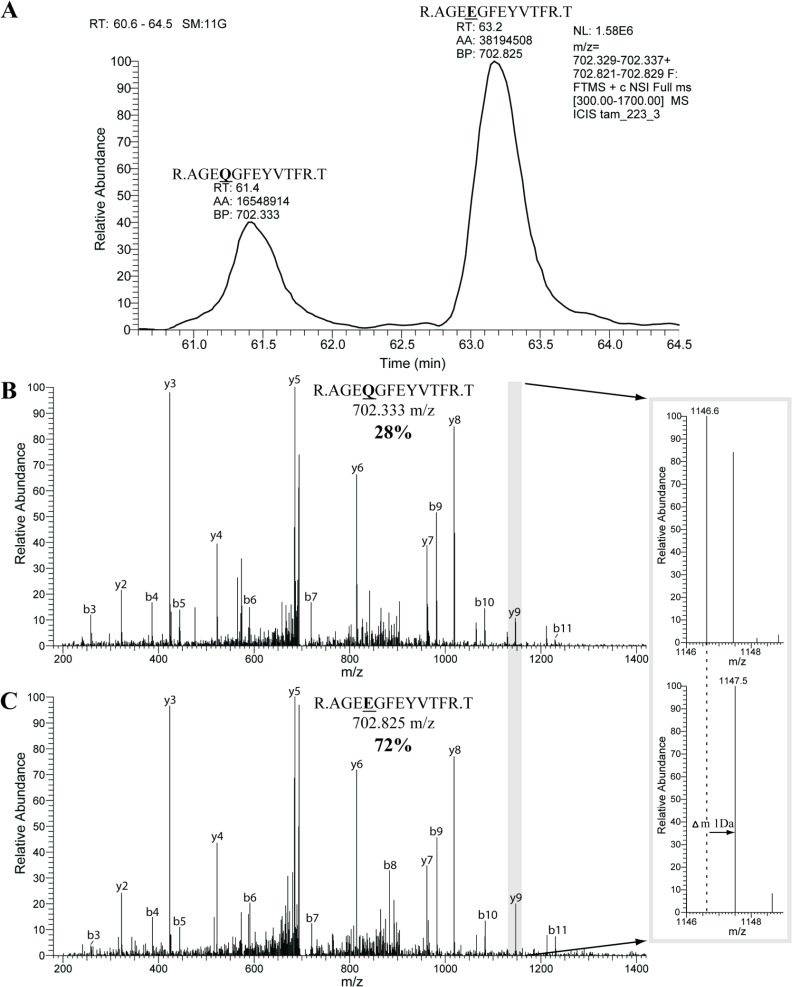
Asparagines and glutamine residues in *S*.*indicum* 11S globulin precursor isoform 3 from lamp 5# were almost fully deamidated. Upper panel: extracted ion chromatogram for native (precursor *m/z* 702.333, z = +2) and deamidated (precursor *m/z* 702.825, *z* = +2) peptide AGQGFEYVTFR from 11S globulin isoform 3 detected in lamp 5# (gel slice #3); RT–retention time, AA–peak integral abundance, BP–base peak. 72% of the peptide was deamidated such that ^9^Gln residue (Q) was converted to glutamic acid ^9^Glu (E) (spectra at the middle and lower panels, respectively). Inset shows the zoomed peaks of fragments y9 whose *m/z* was shifted by 1 Da because of deamidation. The same mass shift was observed for all fragments containing ^4^Glu residue, while masses of other fragments were unchanged.

The second group consisted of seven seed storage proteins from *Sesamum indicum* found in fuel deposits from the lamps 1#a, 2#, 4#, 5#, 6# and 8# (Tables 1 and 2; S1 Dataset). All asparagine and glutamine residues in these proteins were >70% deamidated (Fig 3), which confirmed their ancient origin [32]. At the same time no deamidation was observed in human background proteins and proteins from contemporary reference samples. Considering that neither sesame seeds (whole, ground or milled) nor sesame starch grains were recognized in any of the samples, we hypothesized that these fuel deposits contained sesame oil.

**Table 2:** Proteins from *Sesamum indicum* identified in lamp fuels from Astana necropolis.

No	Protein name	Gene Identifier	MW, kDa	Sequences of matched peptides	Samples from Astana^a^						Contemporary reference samples	
					1#a	2#	4#	5#	6#	8#	Sesame paste (Tahina)^d^	Sesame oil^b^
1	11S globulin seed storage protein 2	gi75315270	51.8	AFYLAGGVPR	+	+	+	+	+	+	+	+
				SPLAGYTSVIR	+	+	+	+	+	+	+	+
				ISGAQPSLR	+	+	+	+	+	+	+	+
				LVYIER	+	+	+	+	+	+	+	
				AGNNGFEWVAFK	+	+		+	+	+	+	
				IQSEGGTTELWDER		+			+	+	+	
				AFDAELLSEAFNVPQETIR		+			+	+	+	
				GLIVMAR		+		+	+	+	+	
				VNQGEMFVVPQYYTSTAR		+				+	+	
				GSQSFLLSPGGR		+				+	+	+
				STIRPNGLSLPNYHPSPR		+				+	+	
				MQSEEEERGLIVMAR		+				+	+	
				READIFSR		+			+			
				AMPLQVITNSYQISPNQAQALKMNR		+						
				AFDAELLSEAFNVPQETIRR		+				**+**	+	
				GSQSFLLSPGGRR		+				**+**		
										**+**20^c^	+30 ^c^	
2	11S globulin precursor isoform 4	gi81238594	52.7	ADIYNPR	+			+	+	+	+	
				FSTINSLTLPILSFLQLSAAR	+	+		+	+	+	+	
				ALMLPAYHNAPILAYVQQGR		+				+	+	
				SFFLAGNPAGR		+			+	+	+	
				GQEQQEYAPQLGR		+		+		+	+	
				GHIITVAR		+			+	+	+	
				GLQVISPPLQR		+			+	+	+	
				EGQVVVVPQNFAVVK		+			+	+	+	
				GLPADVIANAYQISR		+			+	+	+	
				ETMMFSGSFR		+			+	+	+	
				GQHQFGNVFR		+				+	+	
				GLPADVIANAYQISREEAQR		+				+	+	
				EGQVVVVPQNFAVVKR		+				+	+	
				INAQEPTR		+		+	+	+	+	
										+18^c^	+26 ^c^	
3	11S globulin precursor isoform 3	gi81238592	55.3	RGDVLALR		+		+	+	+	+	
				EGVTHWAYNDGDTPIISVSIR		+				+		
				ISTINSQTLPILSQLR		+			+	+	+	
				NGITAPHWSTNSHSALYVTR		+				+	+	
				AGEQGFEYVTFR		+		+	+	+	+	
				AMPDEVVMNAFGVSR		+			+	+	+	
				DEATVFSPGGR		+				+	+	
				SVLNEEVNEGQLVVVPQNFALAIR		+				+	+	
				DVANEANQLDLK					+	+	+	
				LVLPEYGR					+	+	+	
										+17^c^	+28 ^c^	
4	11S globulin	gi13183173	56.6	LTAQEPTIR		+			+	+	+	
				LRENLDEPAR		+				+	+	
				ISSLNSLTLPVLSWLR		+			+	+	+	
				SVFDGVVR		+			+	+	+	
				EGQLIIVPQNYVVAK		+				+	+	
				TNDNAMTSQLAGR	+	+				+	+	
				GLLLPHYNNAPQLLYVVR		+				+	+	
				FQVVGHTGR		+				+	+	
										**+**16^c^	+28 ^c^	
5	7S globulin	gi13183177	67.0	IPYVFEDQHFITGFR						+	+	
				VAILEAEPQTFIVPNHWDAESVVFVAK						+	+	
				INAGTTAYLINR						+	+	
				SFSDEILEAAFNTR						+	+	
				SFSDEILEAAFNTRR						+	+	
				IFGQQRQGVIVK						+		
											+11^c^	
6	Oleosin	gi10834827	17.4	ATGQGPLEYAK						+	+	
				GVQEGTLYVGEK						+	+	
											+5	
7	2S albumin	gi13183175	17.5	QAVRQQQQEGGYQEGQSQQVYQR						+		
				QQQQEGGYQEGQSQQVYQR-						+	+	
								

^a^ found in all repetitive analyses.

^b^ made by traditional cold pressing.

^c^ the number of other peptides matched to the same protein sequence detected in the sample (S1 Dataset).

^d^ another 7 sesame proteins and 109 plant cross-species matches were identified in Tahina sample (S1 Dataset).

We next asked if proteins typical for sesame seeds could be present in sesame oil? To this end, we extracted proteins from a sample of contemporary sesame oil made by traditional cold pressing and also from milled seeds. 11S globulins, which were most abundant proteins in a sesame seeds paste were also found in the oil and exactly the same proteins were detected in Astana fuels (S1 Dataset). To make sure that Astana oil fuels were not produced from other plants, including plants with yet unknown genomes (*e*.*g*. domestic flax or hemp), we collected MS/MS spectra that remained unmatched upon MASCOT searches, subjected them to automated *de novo* interpretation and submitted all candidate sequences to MS BLAST sequence-similarity searches [16,33]. However, no further plant proteins were hit.

**Fig 4:**
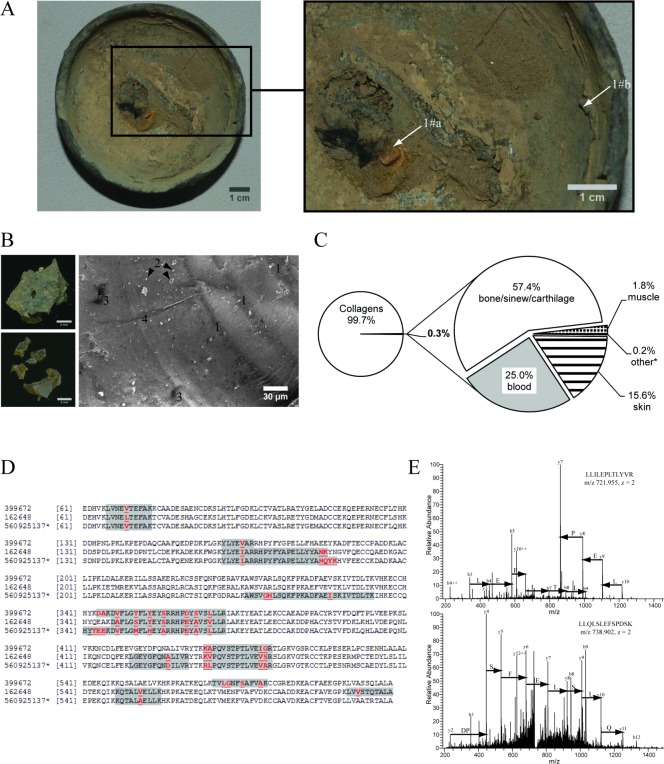
Composition of the organic deposit from lamp 1#. Panel A: top view of the lamp pottery 1#. Deposits 1#a and 1#b were sampled at the designated locations. Brush traces are seen at the right upper corner. Panel B: Light microscopy images of the sample 1#b collected at the edge of lamp 1# (at the left hand side) and electron microscopy image of its inner structure (at the right hand side). Further labeled at the image: (1)—dark and light layers indicate that oil soot and bone glue were intensely mixed; (2) soot and /or mineral additive; (3)—cavities; (4)–trace left by cutting with scalpel. Panel C: Protein composition of the sample 1#b. Panel D: Sequence alignment of serum albumins from *Bos taurus* (gi162648), *Equus caballus* (gi399672) and *Camelus ferus* (gi560925137). Peptides detected in the sample 1#b are highlighted; amino acid residues unique for each sequence are in red. “*”–peptides also matched the sequence of donkey albumin. Panel E: MS/MS spectra of tryptic peptides from *Equidae* surfactant protein latherin detected in 1#b. The spectra matched latherin sequences from various *Equidae* species (*E*.*caballos* (gi 126722737), *E*. *hemionus onager* (gi 58531902) and also to donkey (gi 58531904)).

Protein composition of the sample from lamp 1# strongly differed from other fuels (S1 Dataset). Black soft material (1#a) collected near the wick was identified as a charred sesame oil (Fig 4A; Tables 1 and 2; S2, S3, S4 and S5 Figs; S1 Dataset). Contrary, a glass-like water-soluble protein-rich substance found at the edge of the pottery (1#b) consisted of animal collagens and a small amount of bone, tendon and cartilage proteins—mimecan, lumican, biglycan, decorin *etc* (Fig 4B and 4C; S2, S3, S4 and S5 Figs; S1 Dataset). Altogether, it resembled the composition of typical bone collagen glue [34]. Blood proteins and fat identified in 1#b could originate from bones interior enriched in both adipose and red blood cells (red and yellow marrows). Proteins identified in this sample originated from three domestic animals: camel, cattle and horse (or donkey) (S1 Dataset; Fig 4D). Surfactant latherin and major allergen Equ c1 specific for *Equidae* sweat and dander [35,36], along with skin keratins (Fig 4E, S1 Dataset) suggested that, for making a glue, horse or donkey hide was mixed with raw bones.

Sample 8#, in addition to sesame and *Caprinae* blood proteins, contained two *Bovidae* milk caseins whose asparagine and glutamine residues were strongly deamidated (S1 Dataset). At the same time, we detected no milk whey proteins that used to be a common and readily identifiable component in a variety of contemporary and ancient dairy products, including butter [17] (S1 Dataset). We therefore attributed the fuel in sample #8 to a ghee—clarified cattle butter [37]. This assignment was also consistent with its TAG profile (Fig 2) matching a degraded cattle dairy fat. Although the protein content in a fresh butter is much lower than in other dairy products (*ca* 1% in commercial butter versus *ca* 60% in fresh milk) [38], proteomics readily identified caseins and butyrophilin—but not milk whey proteins that are common to a butter clarified by traditional household recipe (S1 Dataset).

Altogether, proteomics unequivocally established the origin of eight out of the total of nine fuel residues (except 3# that was only attributed to a ruminant adipose fat), including mixed fuels #2 and 8#. It also identified collagen glue in lamp 1#, whose assignment by lipid analysis was ambiguous.

### Wick fibers consisted of hemp, ramie and cotton in mixed yarns

FT-IR analyses identified best preserved wicks in 1#, 2#, 5#, 7# and 8# as plant fibers (S6 Fig). Microscopy and drying-twist test [23] recognized bast fibers derived from three fiber crops: hemp (*Cannabis indica Lam*.), ramie (*Boehmeria nivea L*.) and cotton (*Gossypium sp*.) (S5 Fig; Table 1). Fibers from different plants were twisted together in mixed yarns: ramie with hemp (2#, 7#) or cotton (8#) and cotton/hemp (1#).

## Discussion

Charred fuel deposits and wicks of Astana lamps provided unique material evidence on household, trade and cultural communications of Gaochang settlement in antiquity, which complemented historical writings and archaeological artifacts. Although Gaochang economy was typical for an oasis settlement, it was strongly influenced by its location at the junction of major Silk Road routes.

Hemp fibers, animal adipose fat and animal glue were all produced from locally available raw materials. Domestic hemp was cultivated in Turpan region under the Tang rule as a fiber crop for fabricating textiles and shoes, for decorative purposes, as medicinal plant and oilseed [23,39–41]. Sheep herding was typical in Turpan since antiquity, as corroborated by ancient writings (*e*.*g*. Han Shu book (36-110AD)) and also by faunal remains [42]. Stationing pack animals—horses, donkeys and camels [43,44]—was also common for a big trading hub. Bones and hides of domestic animals were used to afford the glue identified in lamp 1#, which demonstrates rational, skilled and exhaustive exploitation of valuable livestock resources by oasis inhabitants.

We identified the deposit in lamp 1# as an oil soot ink. Traces along the rim of the lamp dish likely left by a brush also supported this notion (Fig 4A). Animal glue mixed with charred organic materials (burned pine) was commonly used in ancient China for fabricating traditional black ink used for calligraphy and brush painting that also persists in many East Asian cultures till present. It is likely that charred ink replaced mineral inks already before the first millennium BC. However, its earliest samples were only found at painted artifacts and therefore their composition was not thoroughly examined. The only known recipe of oil soot ink was mentioned in later historical writings from Northern Song Dynasty (960-1127AD) [45]. To the best of our knowledge the deposit from lamp 1# dated to the first half of the 7^th^ century AD is the earliest sample of oil soot ink stock. We speculate that freshly prepared ink was used by an ancient artist for mural paintings in Astana tombs. Along with sesame seed proteins we detected only traces of glue around the wick in lamp 1# and also in the deposit of lamp 6#, which was excavated without a wick (Table 1). It is therefore conceivable that an unknown artist pushed aside the unburned wick fibers, which might have spoiled continuously drawn lines.

Ramie, cotton and sesame used for making fuels and wicks in Astana, are not domestic to the Turpan region. Ramie fibers in mixed yarns 2#, 7# and 8# (Table 1) could be threads from outworn garments brought as a merchandise from southern China where this plant is native [46]. Cotton remained a rare commodity in China until 9^th^ century AD [47], yet according to Liang Shu historical book it was probably planted in Gaochang already in the 6^th^ century AD.

The discovery of sesame oil in Astana lamps is particularly important for understanding the history of expansion and exploitation of *Sesamum indicum*, one of the major economic crops of Eastern Eurasia today [48]. Although it was cultivated as an oilseed in South Asia since as early as 2000BC [49], sesame started its eastward spread *via* the Silk Road and likely reached China shortly before the first millennium AD. According to historical writings flavorful sesame seeds were used as an exotic condiment in Eastern Han Dynasty (25-220AD) [50], although interpretations of ancient Chinese might be ambiguous because the word “huma” (胡麻, hu = foreign, ma = fiber) equally refers to sesame and flax. There is no material evidence if sesame was commonly cultivated in Tang China and its seeds are not included in the inventory of plants recovered in 4–8 century AD burials of Astana [50,51]. Even less is known about its early exploitation as an oilseed and if sesame oil was utilized in ancient China. Yet, sesame oil in Astana lamps indicated that it was already used as a lamp fuel in the northwestern China as early as the first half of the 7^th^ century AD.

We argue that the use of sesame oil as a fuel, particularly at the burial sites, could coincide with changing of ritual practices of Gaochang population. Sesame oil is essential for Buddhist rites and Buddhism reached Turpan region *via* Silk Road during first centuries AD. It played a major role in spiritual life of Gaochang community under Tang rule [52,53]. It is therefore conceivable that also later the tradition of sesame agriculture followed Buddhism expansion to Eastern Asia. Conceivably, a cattle ghee found in the lamp 8# was also associated with funeral practices of the local Buddhist followers. First mentioned as a sacred illuminant in Rig Veda–the collection of Vedic Sanskrit hymns composed around 1500 BC [54], ghee was used in religious rites and fire worshiping in Hinduism and Buddhism. Gaochang population might also took advantage of the dietary properties and assumed curative powers of a clarified butter [55] and sesame oil [56]. Furthermore, their extended shelf life was well suiting seasonal nutrition habits of desert oasis inhabitants.

We also concluded that proteomics methods that were only recently applied for the characterization of charred archaeological deposits [16,17,34,57] could unequivocally identify the organismal origin of ancient oil and fat illuminants. Proteomics could discern the molecular composition of complex protein mixtures and is much less affected by massive contaminations with organic substances of fungi, bacteria or even human origin, which constitutes a serious bottleneck for methods relying on matching molecular profiles or isotopic analyses.

## Supporting information

S1 Dataset Proteins identified in Astana samples and contemporary oils and fats by GeLC-MS/MS. (XLS)

S2 Dataset TAG species quantified by shotgun lipidomics in Astana fuels and contemporary oils and fats. (XLS)

S1 Fig TAG profiles of contemporary adipose and diary fats. (PDF)

S2 Fig Light microscopy images of deposits from Astana lamps. (PDF)

S3 Fig Electron microscopy images of the surface and inner structure of lamp deposits. (PDF)

S4 Fig Deposits images acquired by polarized light microscopy. (PDF)

S5 Fig Light microscopy images of fibers from wicks of Astana oil lamps. (PDF)

S6 Fig FTIR spectra of lamp deposits and wicks. (PDF)

## Abbreviations

FT-IR: Fourier Transform infrared spectrometry
FT MS: Fourier Transform mass spectrum
FT MS/MS: Fourier Transform tandem mass spectrum
GC-MS: gas chromatography–mass spectrometry
LC-MS/MS: liquid chromatography—tandem mass spectrometry
SDS: sodium dodecylsulfate
TAG: triacylglycerols
