# Supplementary material for: Open sesame: Identification of sesame oil and oil soot ink in organic deposits of Tang Dynasty lamps from Astana necropolis in China
Source: PLoS One. 2017 Feb 24;12(2):e0158636. doi: 10.1371/journal.pone.0158636 (PMC5325208; doi:10.1371/journal.pone.0158636)
Supplement: S1 Fig — (PDF) [file pone.0158636.s003.pdf]

Supplementary Figure 1S: Modern adipose and dairy fat references

Sample	MS profile	42:0 / 44:0	46:0	Source
	**Modern adipose**			
pig	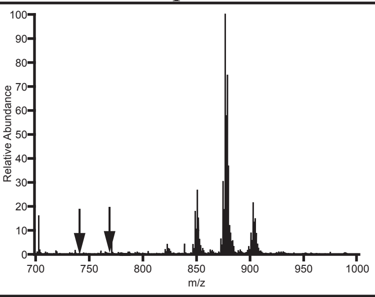	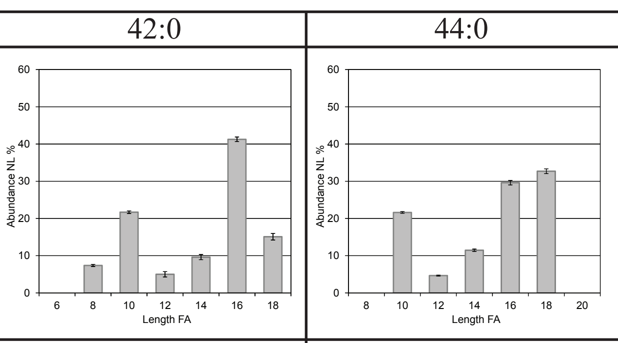	[10-18]	Vorwerk Podemus Biofleisch- und Wurstwaren Vera und Bernhard Probst GbR
sheep	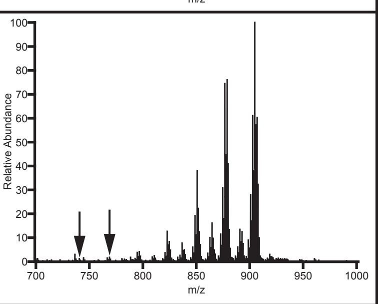	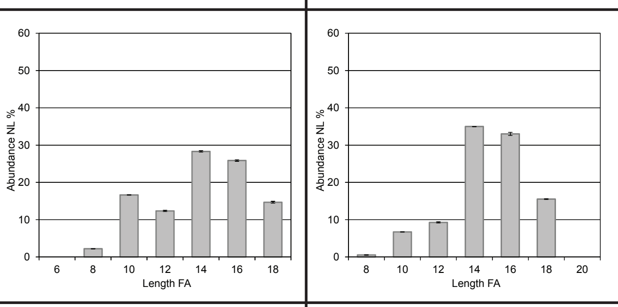	[10-18]	Exporter: New Zeeland
calf	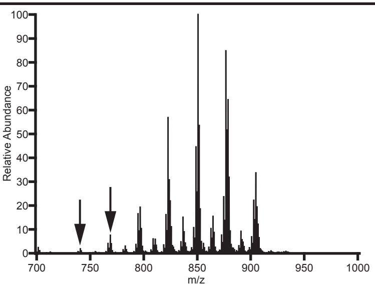	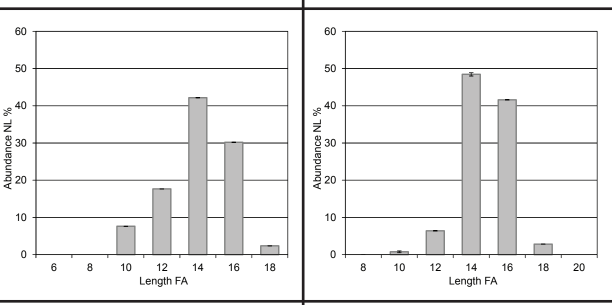	[14-18]	Local butcher, Dresden, Germany
cow	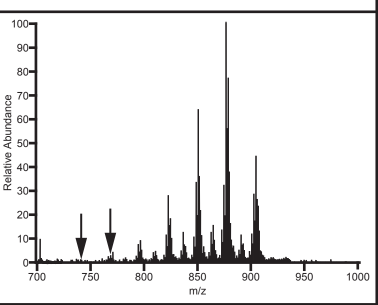	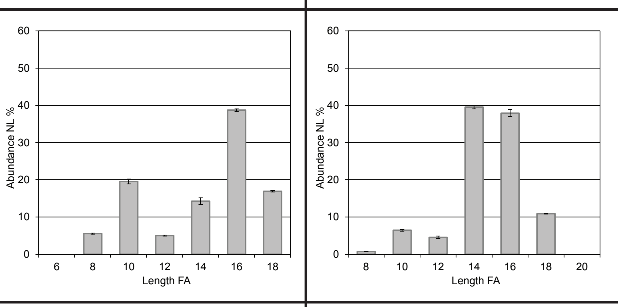	[14-18]	Vorwerk Podemus Biofleisch- und Wurstwaren Vera und Bernhard Probst GbR
yak	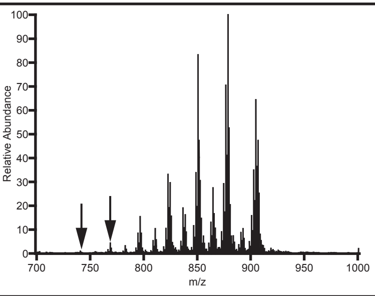	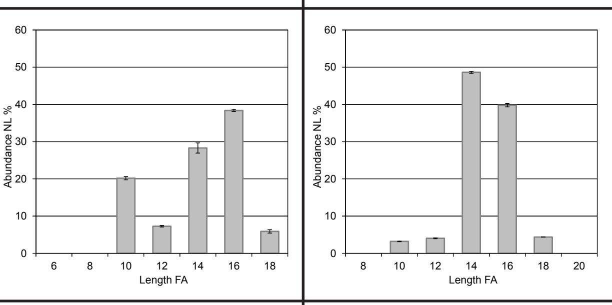	[14-18]	private vendor, China
horse	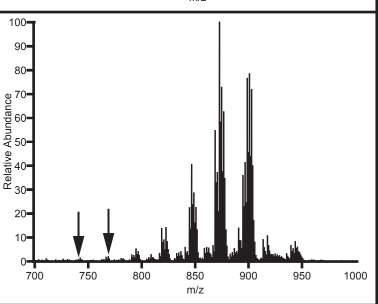	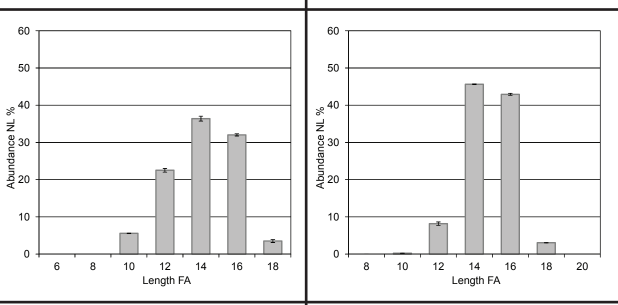	[14-18]	private vendor, China
camel	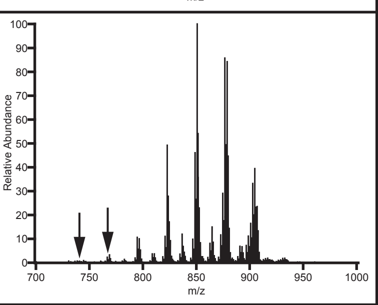	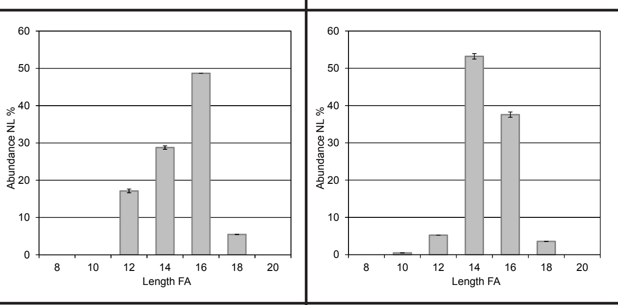	[14-18]	private vendor, China
goat	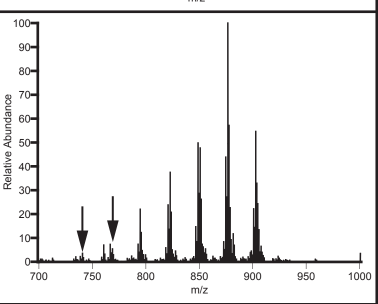	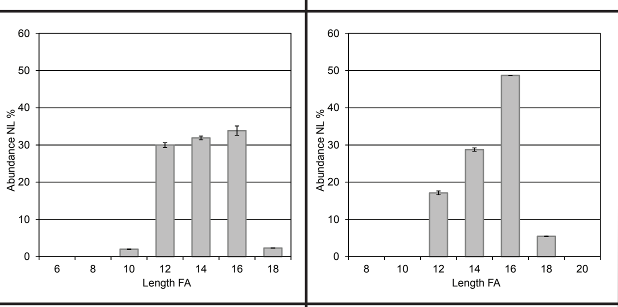	[12-18]	Local butcher, Berlin, Germany
deer	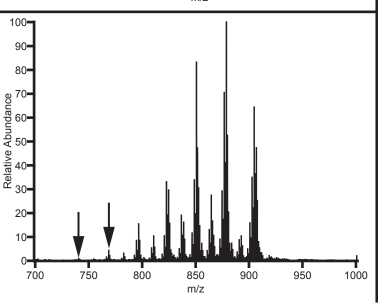	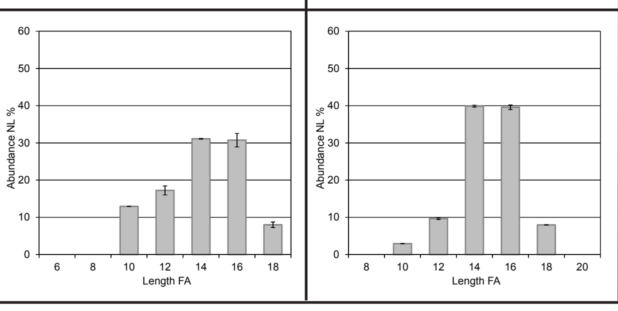	[14-18]	Local butcher, Dresden, Germany
	**Modern dairy products**			
yak milk	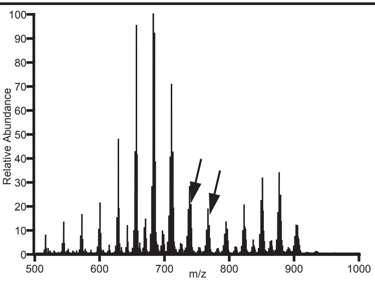	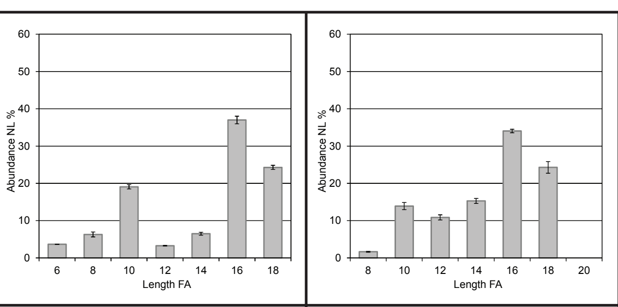	[10-18]	private vendor, China
ewe milk	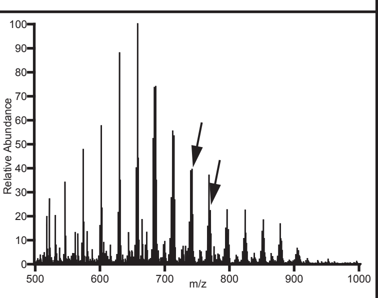	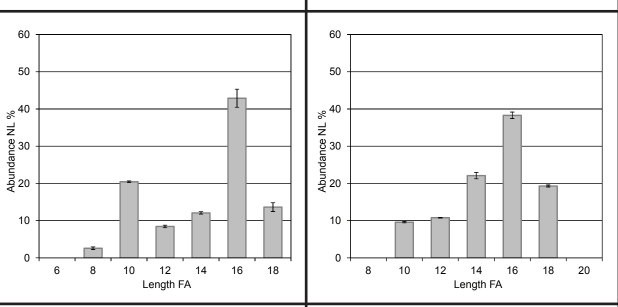	[10-18]	Reutebachhof, Geifertshofen, Maisch GbRa
mare milk	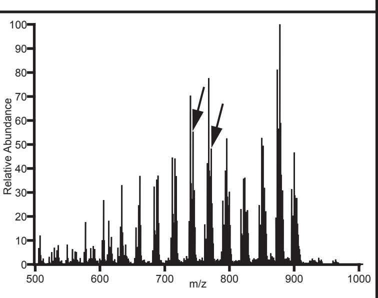	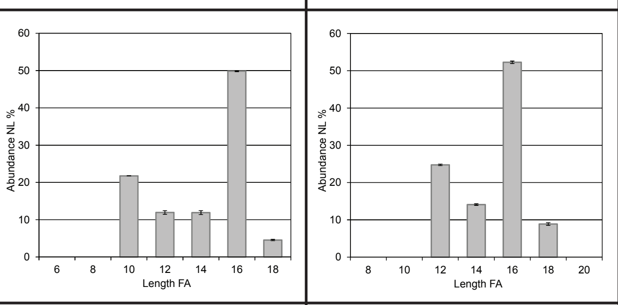	[12-18]	private vendor, China
cow butter, cow milk	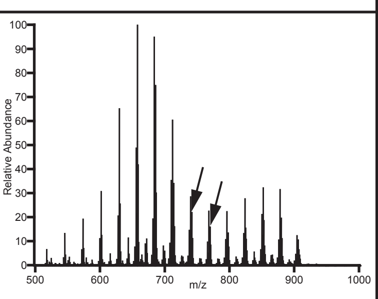	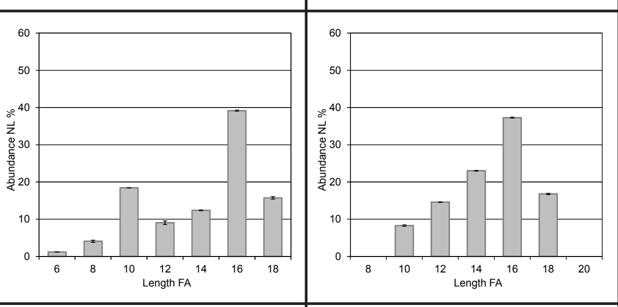	[10-18]	IDB GmbH, Neukirchen-Vluyn, Molkerei SÖBBEKE GmbH
goat milk	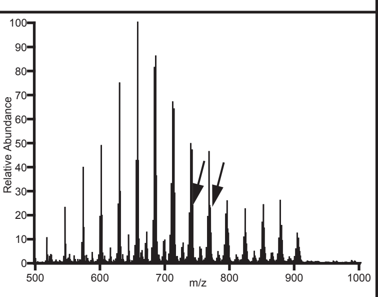	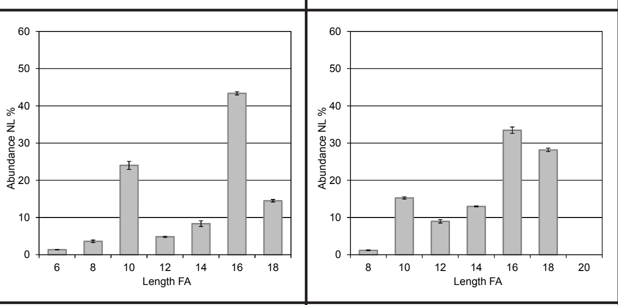	[10-18]	Andechser Molkerei Scheitz GmbH
camel milk	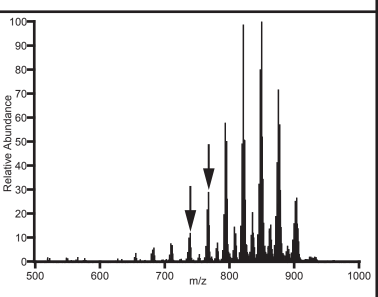	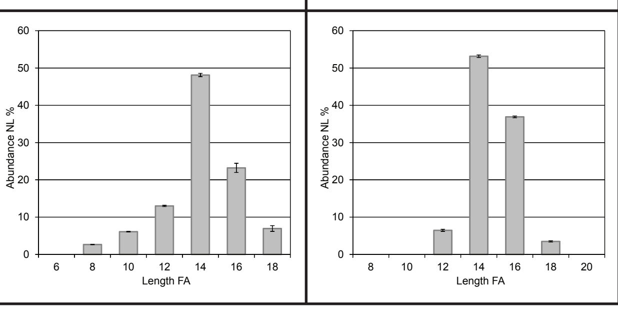	[12-18]	private vendor, China
	**Modern vegetable oil**			
sesame oil	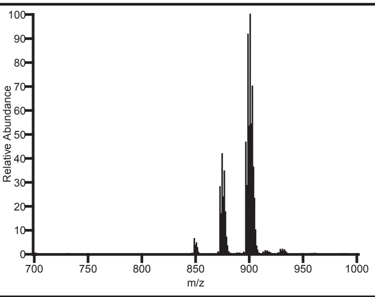	n.d. / n.d.	n.d.	Willys Weise Erben Ölmühle, Oberbobritzsch

Figure 1S: Modern adipose and dairy fat references."MS profile" column shows full ms-spectra of TAGs in the corresponding sample; TAGs 42 and 44 are designed with arrows. Columns "42:0" and "44:0" show diagrams of relative abundance of fatty acid moieties in TAG 42:0 and TAG 44:0 averaged from two sample measurements; n.d stays for non-identified; Column "46:0" reports the numbers of carbon atoms in fatty acid moieties of TAG 46:0.
