# Supplementary material for: Open sesame: Identification of sesame oil and oil soot ink in organic deposits of Tang Dynasty lamps from Astana necropolis in China
Source: PLoS One. 2017 Feb 24;12(2):e0158636. doi: 10.1371/journal.pone.0158636 (PMC5325208; doi:10.1371/journal.pone.0158636)
Supplement: S2 Fig — (PDF) [file pone.0158636.s004.pdf]

Supplementary Figure 2S . Light microscopy of deposits from Astana lamps

1#a

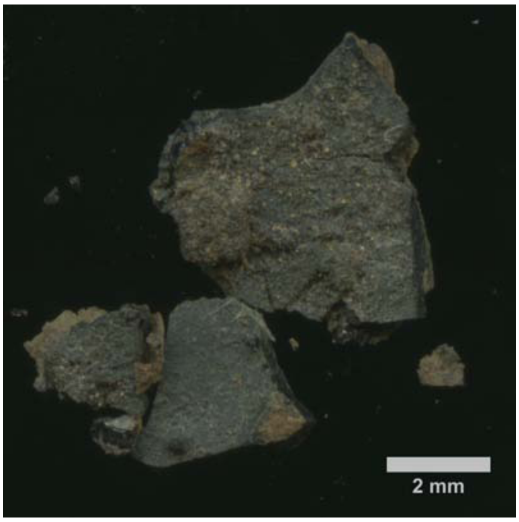

4#

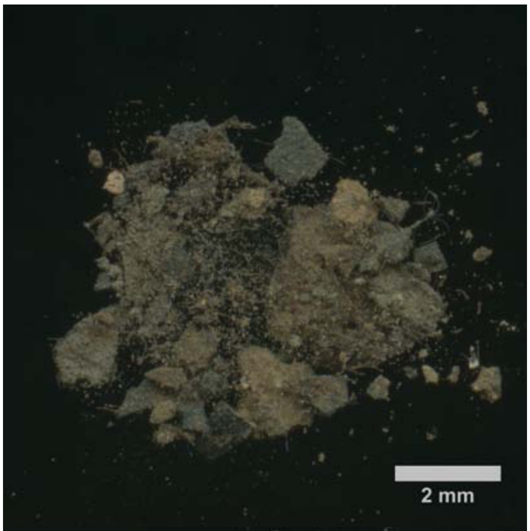

1#b

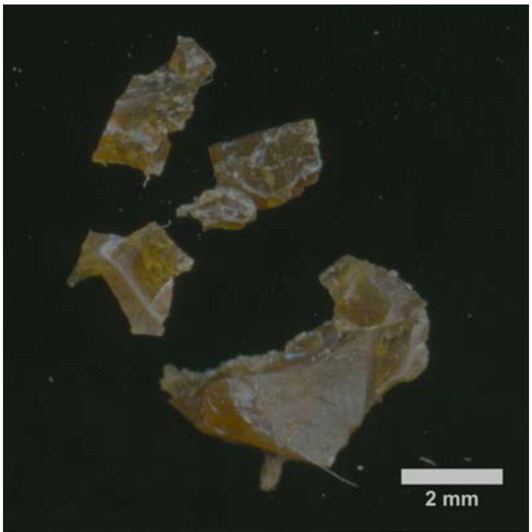

5#

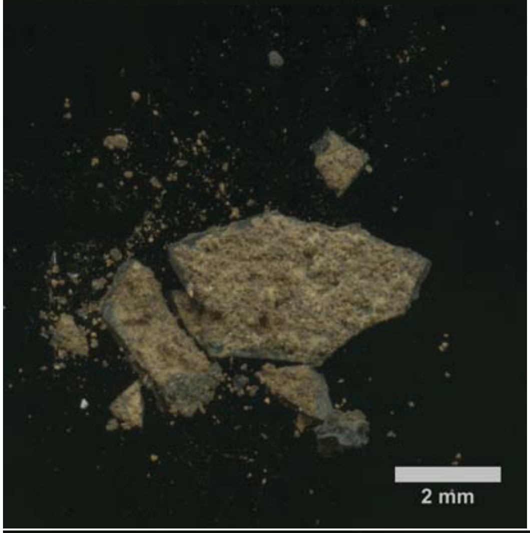

2#

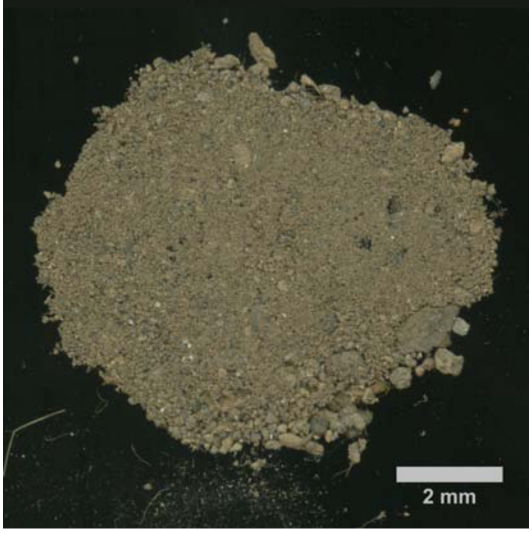

6#

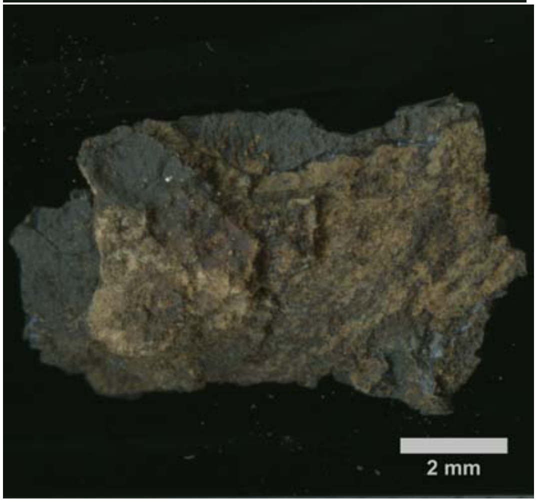

3#

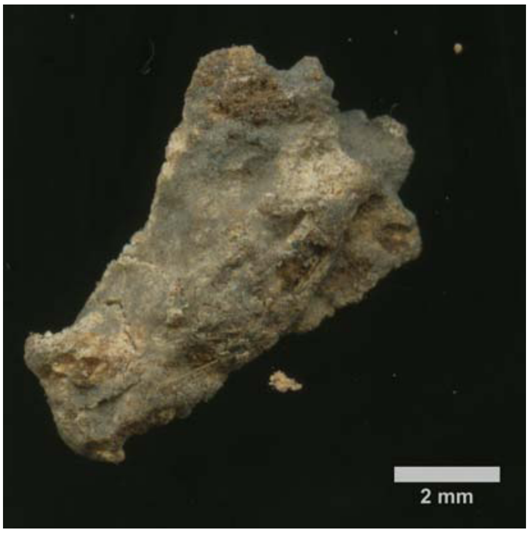

7#

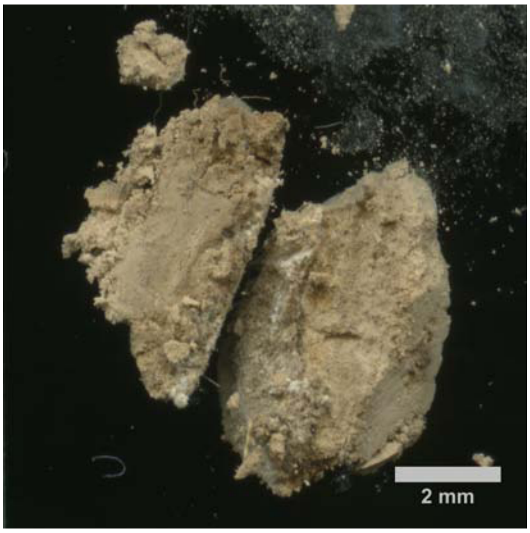

Figure 2S: Light microscopy of deposits from Astana lamps. Ancient materials had yellowish to black color and different density and texture - from soft easily crumbled by pressing with fingers (as 2# and 3#) to hard with sharp edges (as #1b).
