# Supplementary material for: Open sesame: Identification of sesame oil and oil soot ink in organic deposits of Tang Dynasty lamps from Astana necropolis in China
Source: PLoS One. 2017 Feb 24;12(2):e0158636. doi: 10.1371/journal.pone.0158636 (PMC5325208; doi:10.1371/journal.pone.0158636)
Supplement: S3 Fig — (PDF) [file pone.0158636.s005.pdf]

Supplementary Figure 1S B: Electron microscopy of surface and inner structure of Astana dried residues.

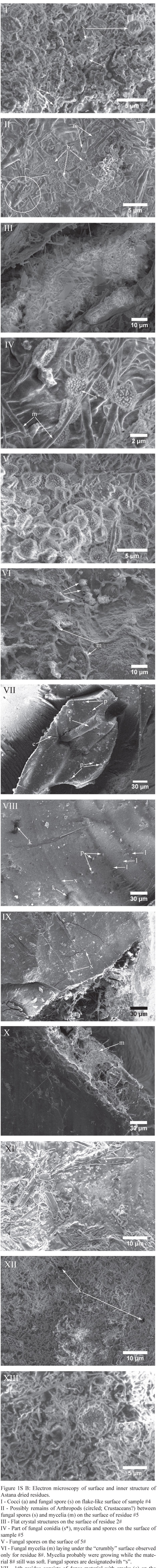

Figure 1S B: Electron microscopy of surface and inner structure of Astana dried residues.

I - Cocci (a) and fungal spore (s) on flake-like surface of sample #4

II - Possibly remains of Arthropods (circled; Crustaceans?) between fungal spores (s) and mycelia (m) on the surface of residue #5

III - Flat crystal structures on the surface of residue 2#

IV - Part of fungal conidia (s*), mycelia and spores on the surface of sample #5

V - Fungal spores on the surface of 5#

VI - Fungal mycelia (m) laying under the "crumbly" surface observed only for residue 8#. Mycelia probably were growing while the material 8# still was soft. Fungal spores are designatedwith "s".

VII - 1#b residue consists of dense material with cracks (c) on the smooth almost fungi-free surface and includes small particles of unknown nature (p). A fiber (f) probably from wick could be seen incorporated into 1#b matrix.

VIII - section of 1#b residue: dark and light layers (l), incorporated particles (p), cavities (k), trace of cutting scalpel (x)

IX - Inner cracks (c) on a section view of dense material of residue 6#; similar structures were observed for residues 1a#, b#, 4#, 5#. Fungal mycelia (m) lays on the surface; cutting scalpel traces (x)

X - fungal mycelia (m) lays free on the surface of black residue 6#

XI - Debris of unknown nature on the surface of 2#

XII - "Folded" surface of residue 7# with a few micron cavities (k)

XIII - 7# unknown structure on the surface of the sample
