# Supplementary material for: Open sesame: Identification of sesame oil and oil soot ink in organic deposits of Tang Dynasty lamps from Astana necropolis in China
Source: PLoS One. 2017 Feb 24;12(2):e0158636. doi: 10.1371/journal.pone.0158636 (PMC5325208; doi:10.1371/journal.pone.0158636)
Supplement: S4 Fig — (PDF) [file pone.0158636.s006.pdf]

Supplementary Figure 4S : Polarized light microscopy

	transmissive	polarized
#4	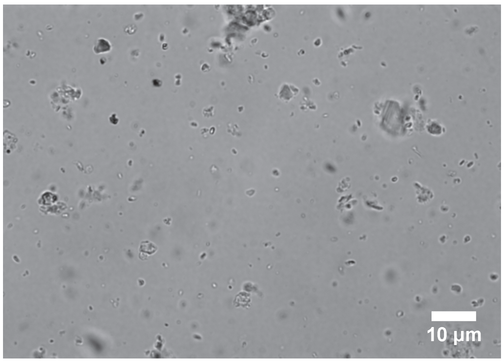	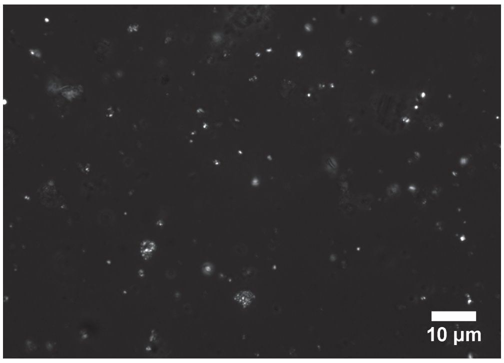
#5	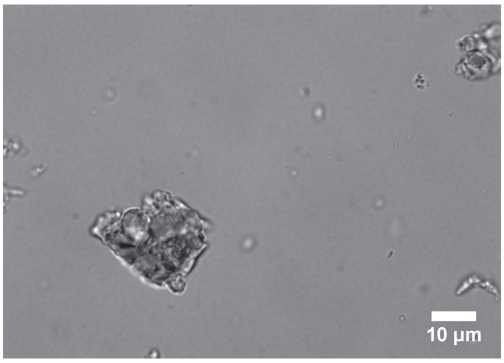	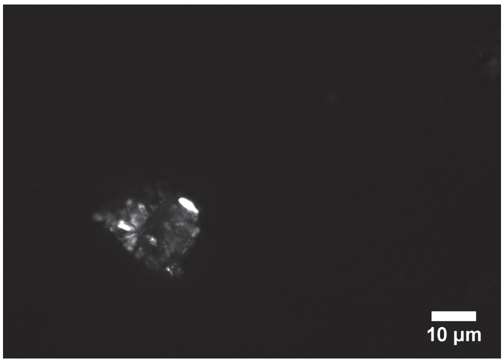
modern sesame	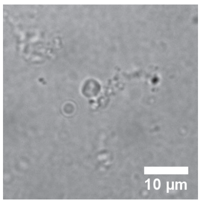	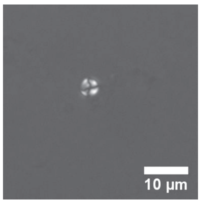
modern barley	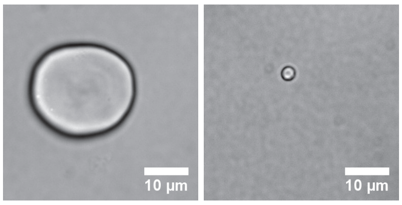	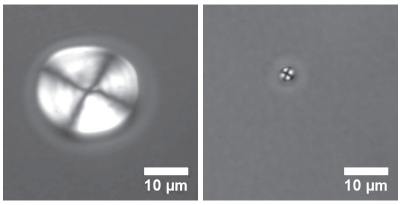

Figure 4S: Polarized light microscopy. Samples #4 and #5 are shown as typical examples. ~1-15 um particles of a mineral material are seen under polarized light, however no starch grains were observed . Starch grains from modern sesame and barley are shown for comparison.
