# Supplementary material for: Open sesame: Identification of sesame oil and oil soot ink in organic deposits of Tang Dynasty lamps from Astana necropolis in China
Source: PLoS One. 2017 Feb 24;12(2):e0158636. doi: 10.1371/journal.pone.0158636 (PMC5325208; doi:10.1371/journal.pone.0158636)
Supplement: S5 Fig — (PDF) [file pone.0158636.s007.pdf]

Supplementary Figure 5S

Microscopic analysis of fibers from wicks of Astana oil lamps

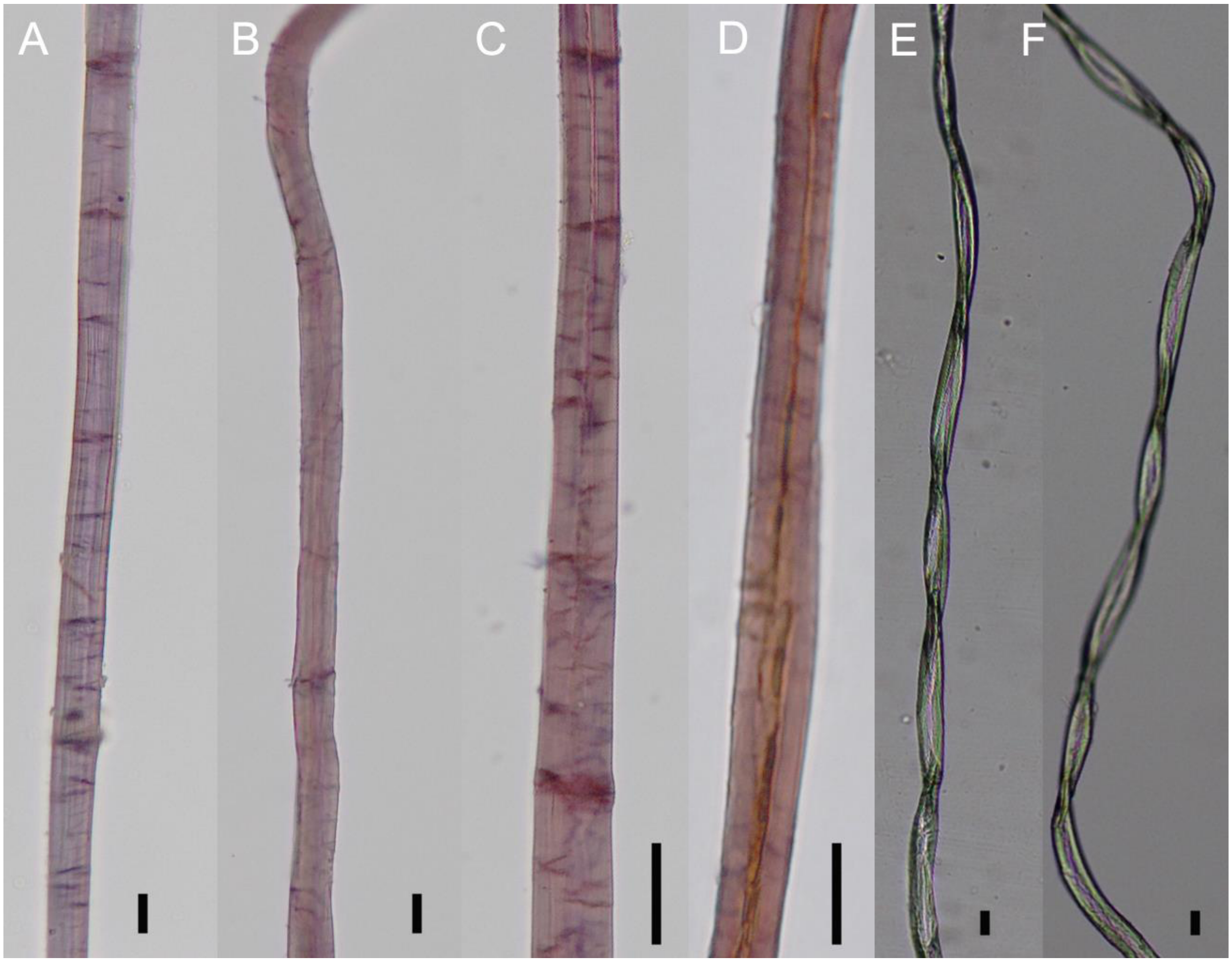

Microscopic structure of wick fibers in Astana lamps and corresponding references.
(A) Modern hemp blast fiber (Cannabis indica Lam.) and **(B)** hemp fiber from wick 1# (as well as 2# and 7#; data not shown) showed rough surface with cross-markings, longitudinal striations and rotated *counterclockwise* in the drying-twist tests.
(C) Modern ramie blast fiber (Boehmeria nivea L.) and **(D)** ramie fiber from wick 2# (as well as 7# and 8#; data not shown) have longitudinal striations on their surface, but rotated *clockwise* in the drying-twist tests.
(E) Modern cotton blast fiber and **(F)** cotton fiber from wick 8# (and also 1#; data not shown) are flat and crimping without transverse stripes.
