# Supplementary material for: Open sesame: Identification of sesame oil and oil soot ink in organic deposits of Tang Dynasty lamps from Astana necropolis in China
Source: PLoS One. 2017 Feb 24;12(2):e0158636. doi: 10.1371/journal.pone.0158636 (PMC5325208; doi:10.1371/journal.pone.0158636)
Supplement: S6 Fig — (PDF) [file pone.0158636.s008.pdf]

Supplementary Figure 6S. FTIR analysis of Astana residues.

A. Ancient fuels

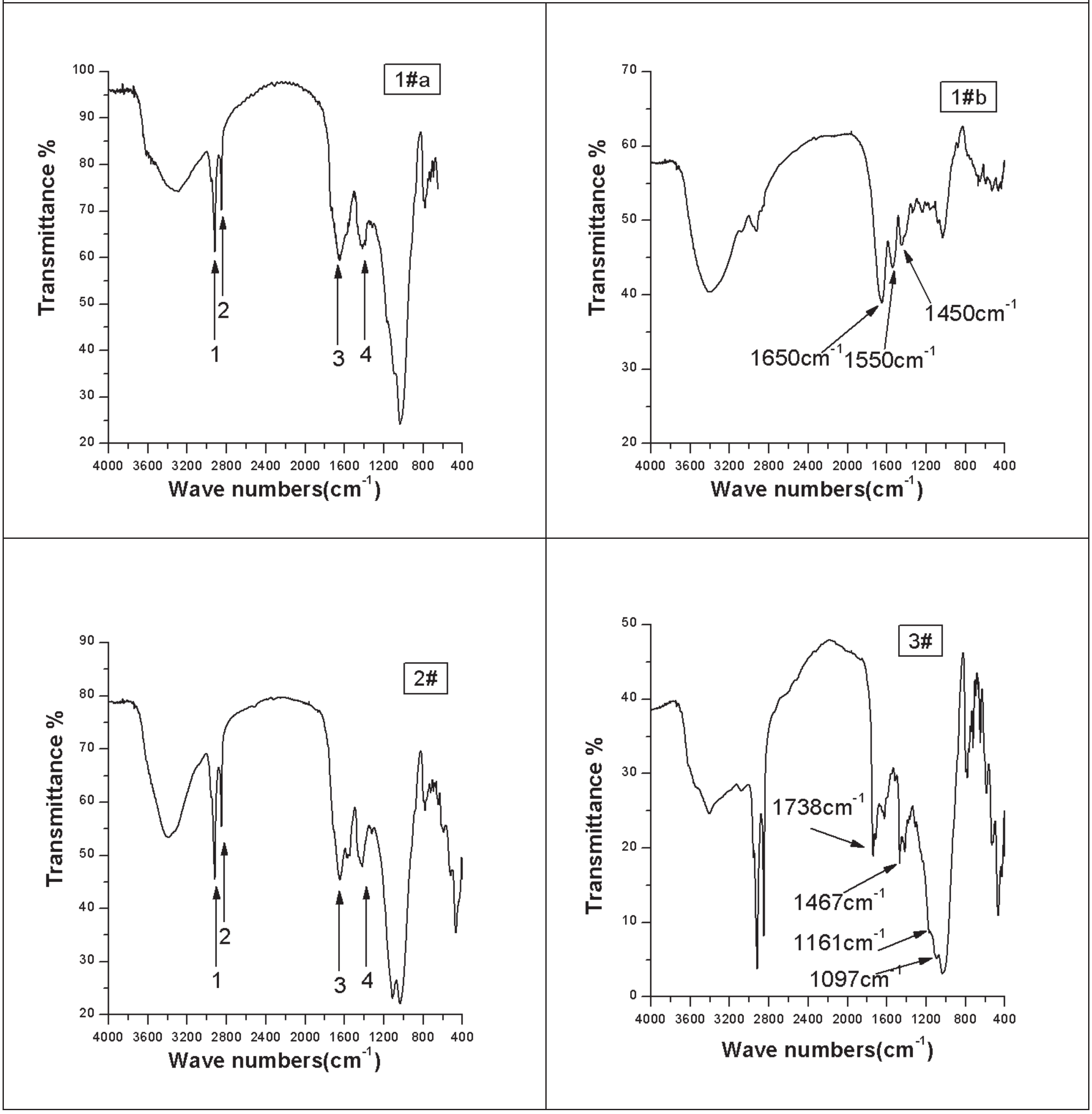

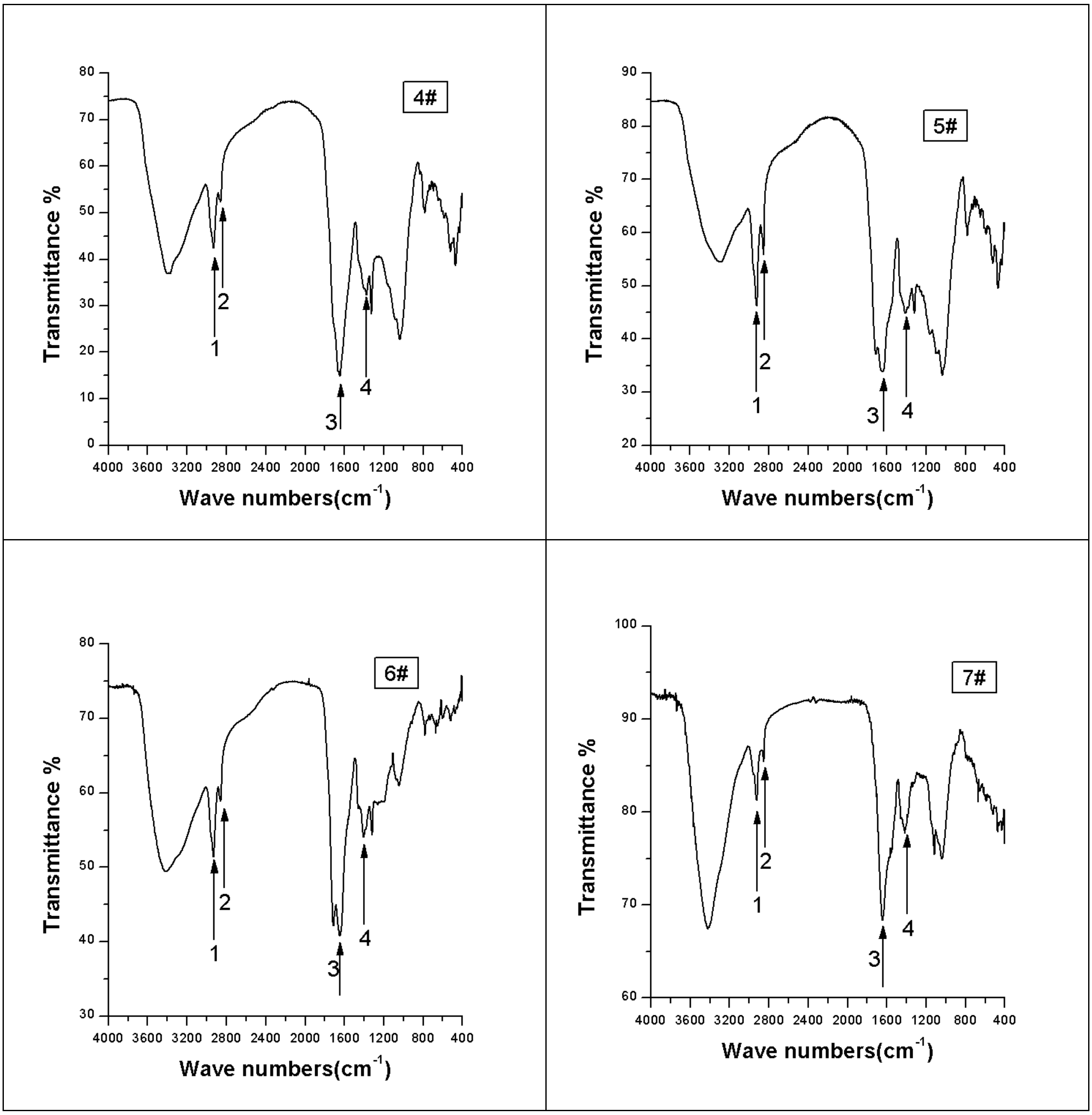
4#
5#
6#
7#
Transmittance %
Wave numbers(cm-1)
4000 3600 3200 2800 2400 2000 1600 1200 800 400
1
2
3
4

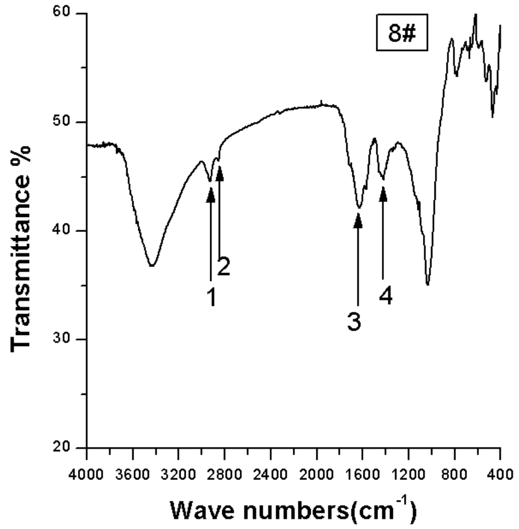

B. Ancient wick fibers

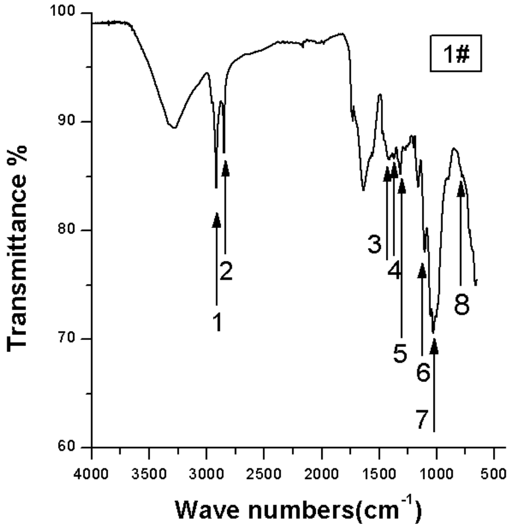

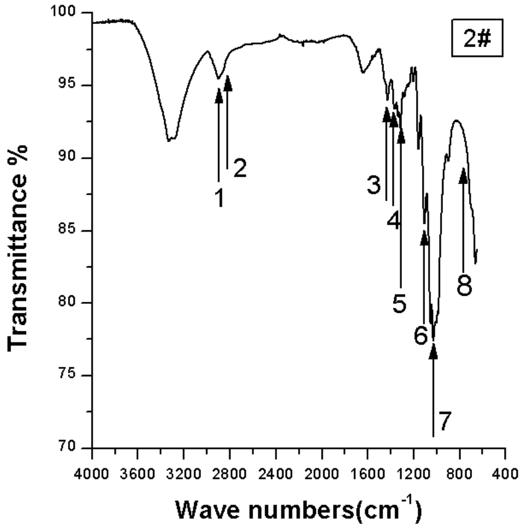

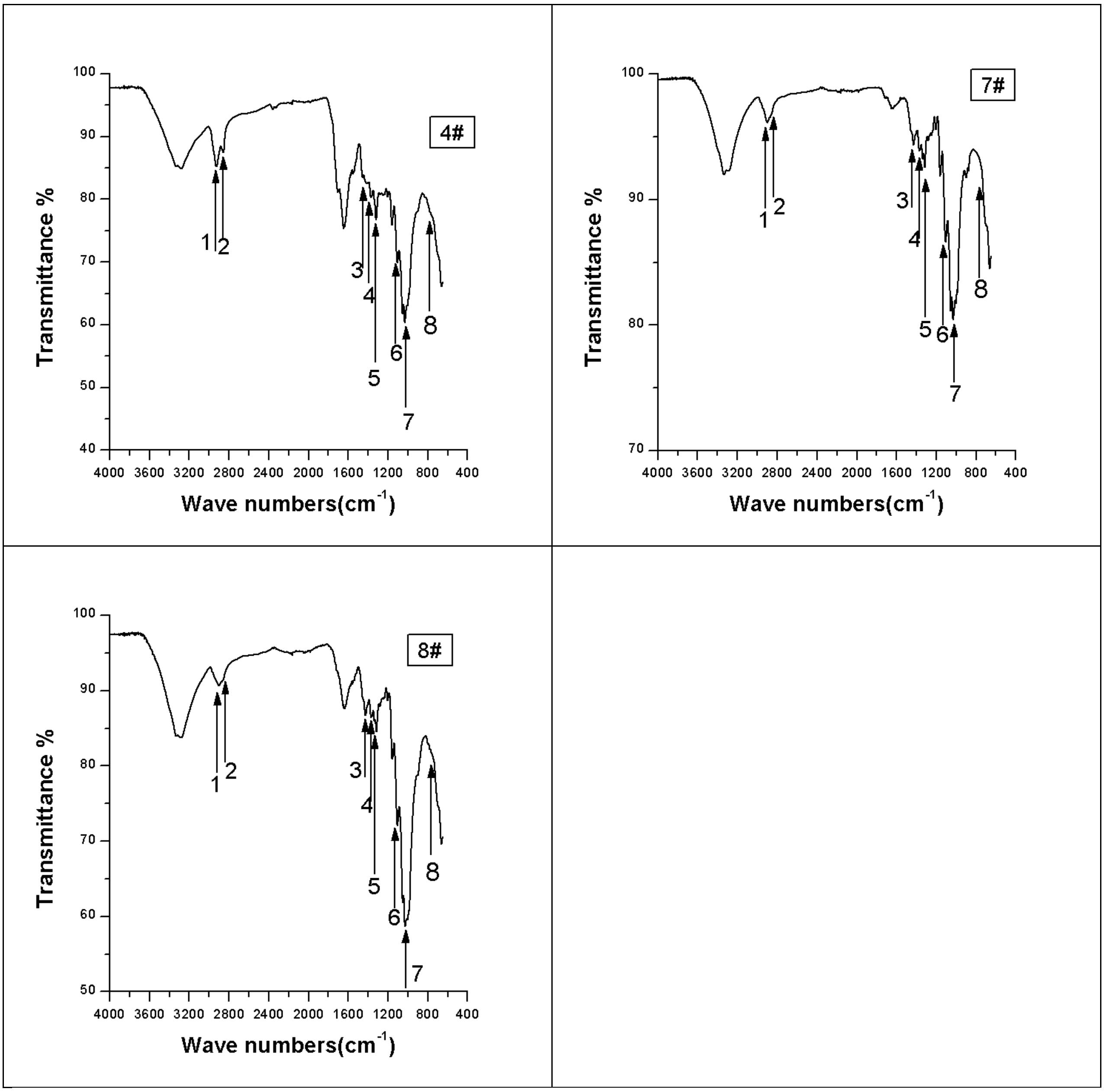

A: Ancient fuels. Spectrum of sample 1#b shows protein-characteristic amid signals: amide I band at 1650 cm^{-1}, amide II band at 1550 cm^{-1} and amide III band at 1450 cm^{-1}.
Spectrum of sample 3# shows fat-typical signals at 1738 cm^{-1}, 1467 cm^{-1}, 1161 cm^{-1} and 1097 cm^{-1} corresponding respectively to ester group in triacylglycerol, bending vibrations of the CH2 and CH3 aliphatic groups, stretching vibrations of the C-O group in esters and C-H bending vibrations of fatty acids. Spectra of samples 1#a, 4#, 5# and 6# look similar and present signals typical for organic material burnt to different degree (Oudemans, Boon et al. 2007).
B: Ancient wick fibers. All Astana wick fibers show characteristic cellulose peaks at 2920, 2851, 1417, 1370, 1317, 1115, 1037 and 779 cm^{-1} (Liu 2010).
